# MDP-based Cleaner Restores the Bond Strength and Chemical Interaction of a Self-adhesive Resin Cement to Blood-contaminated Zirconia

**DOI:** 10.3290/j.jad.c_2865

**Published:** 2026-11-13

**Authors:** Pharsiri Sirirangsee, Kittisak Sanon, Pasiree Thongthai, Janeta Chavanavesh, Irin Chiwasuchin, Junji Tagami

**Affiliations:** a Pharsiri Sirirangsee Postgraduate Student, Department of Operative Dentistry, Faculty of Dentistry, Chulalongkorn University, Bangkok, Thailand; Smart Innovations for Preventive and Restorative Dentistry Research Group, Faculty of Dentistry, Chulalongkorn University, Bangkok, Thailand. Principal investigator, performed the experiments and certain tests, wrote the manuscript, contributed substantially to the discussion.; b Kittisak Sanon Assistant Professor, Department of Operative Dentistry, Faculty of Dentistry, Chulalongkorn University, Bangkok, Thailand; Smart Innovations for Preventive and Restorative Dentistry Research Group, Faculty of Dentistry, Chulalongkorn University, Bangkok, Thailand. Idea, hypothesis, experimental design, wrote and proofread the manuscript, contributed substantially to discussion.; c Pasiree Thongthai Lecturer, Department of Operative Dentistry, Faculty of Dentistry, Chulalongkorn University, Bangkok, Thailand; Smart Innovations for Preventive and Restorative Dentistry Research Group, Faculty of Dentistry, Chulalongkorn University, Bangkok, Thailand. Idea, hypothesis, experimental design, performed statistical evaluation, wrote and proofread the manuscript, contributed substantially to discussion.; d Janeta Chavanavesh Assistant Professor, Department of Orthodontics, Faculty of Dentistry, Chulalongkorn University, Bangkok, Thailand; Smart Innovations for Preventive and Restorative Dentistry Research Group, Faculty of Dentistry, Chulalongkorn University, Bangkok, Thailand. Idea, hypothesis, resources, wrote and proofread the manuscript, contributed substantially to discussion.; e Irin Chiwasuchin Postgraduate Student, Department of Operative Dentistry, Faculty of Dentistry, Chulalongkorn University, Bangkok, Thailand; Smart Innovations for Preventive and Restorative Dentistry Research Group, Faculty of Dentistry, Chulalongkorn University, Bangkok, Thailand. Performed the experiments and certain tests, wrote the manuscript.; f Junji Tagami Emeritus Professor, Department of Cariology and Operative Dentistry, Graduate School of Medical and Dental Sciences, Institute of Science Tokyo, Tokyo, Japan; Adjunct Professor, Department of Operative Dentistry, Faculty of Dentistry, Chulalongkorn University, Bangkok, Thailand; Smart Innovations for Preventive and Restorative Dentistry Research Group, Faculty of Dentistry, Chulalongkorn University, Bangkok, Thailand. Idea, hypothesis, experimental design, consulted on statistical evaluation, wrote and proofread the manuscript, contributed substantially to discussion.

**Keywords:** MDP-based cleaner, self-adhesive resin cement, shear bond strength, XPS, zirconia

## Abstract

**Purpose:**

Bonding to zirconia remains challenging because of its polycrystalline structure, and intraoperative blood contamination further compromises resin–zirconia adhesion. 10-methacryloyloxydecyl dihydrogen phosphate (MDP)-based cleaners have been proposed to restore bond strength to contaminated zirconia, but their mechanism and durability under aging remain incompletely characterized. This study evaluated the effect of an MDP-based cleaner on the shear bond strength (SBS) and chemical interaction of a self-adhesive resin cement bonded to clean and blood-contaminated zirconia.

**Methods and Materials:**

Zirconia discs were air-abraded and allocated to four groups: clean zirconia without pretreatment; clean zirconia treated with MDP-cleaner; blood-contaminated zirconia without pretreatment; and blood-contaminated zirconia treated with MDP-cleaner. Self-adhesive resin cement was bonded to each surface. SBS was measured after 24-h water storage or 5,000 thermocycles (n = 15) and analyzed with three-way ANOVA and Tukey’s test (α = 0.05). Surface morphology was evaluated by scanning electron microscopy (SEM), and chemical composition and cement–zirconia interaction were evaluated by X-ray photoelectron spectroscopy (XPS).

**Results:**

Blood contamination significantly reduced 24-h SBS (*p* < 0.001), decreasing further after thermocycling (*p* = 0.004). MDP-cleaner treatment recovered SBS on contaminated zirconia (*p* < 0.001) and maintained stability after thermocycling (*p* = 0.12). SEM showed no morphological alterations. XPS showed partial reduction of nitrogen-containing blood proteins and a pattern consistent with chemical interactions between MDP-derived phosphate groups and zirconia.

**Conclusion:**

MDP-based cleaner restored bond strength of self-adhesive resin cement to blood-contaminated zirconia and maintained it after thermocycling. This was accompanied by partial reduction of blood–derived surface contamination and uptake of MDP-derived phosphate onto the zirconia surface, with negligible effect on clean zirconia.

Zirconia ceramics have become a mainstay of contemporary fixed prosthodontics because of their favorable mechanical properties and increasingly acceptable esthetics.^10,13,16
^ However, achieving a durable bond between zirconia and resin-based luting materials remains a persistent clinical challenge. Because zirconia is a polycrystalline ceramic that lacks a glass phase, it cannot be conditioned by the hydrofluoric acid etching and silanization routinely used for silica-based ceramics, and its inherent chemical inertness limits the formation of strong, lasting adhesive bonds.^10,13,16
^


The introduction of self-adhesive resin cements containing 10-methacryloyloxydecyl dihydrogen phosphate (MDP) has substantially simplified and improved resin bonding to zirconia. The phosphate ester group of MDP chemically coordinates with zirconium atoms at the ceramic surface to form stable Zr–O–P bonds, while its methacrylate terminus copolymerizes with the resin matrix.^12^ Combining this chemical mechanism with the micromechanical retention created by alumina air-particle abrasion is now regarded as the standard protocol for zirconia bonding, and has translated into favorable clinical survival rates for resin-bonded zirconia restorations when properly executed.^16,17
^


Despite these advances, debonding remains among the most frequently reported complications of resin-bonded zirconia restorations.^16^ A major contributing factor is intraoperative contamination: the fitting surface of a zirconia restoration is almost inevitably exposed to saliva or blood during clinical try-in, and both fluids markedly reduce resin–zirconia bond strength.^6,21
^ Blood, in particular, deposits a tenacious, protein-rich film onto the roughened, air-abraded zirconia surface.^11,12
^ These adsorbed proteins sterically block the phosphate groups of MDP from reaching the underlying zirconium oxide, and conventional decontamination methods such as water rinsing, alcohol, or phosphoric-acid etching have shown inconsistent efficacy in reversing this effect and can even further compromise bond strength.^6,20
^


MDP-based cleaning solutions have recently been introduced as an alternative decontamination strategy. Rather than relying solely on the alkaline or oxidative chemistry of earlier zirconia cleaners, these products exploit the amphiphilic, surfactant-like structure of MDP itself: the hydrophobic tail is thought to bind and lift organic contaminants away from the ceramic surface, while the liberated phosphate groups simultaneously re-establish chemical coordination with the underlying zirconium oxide.^1,7,8,14,19
^ This dual decontamination-and-bonding mechanism has shown promise for restoring bond strength to saliva- and blood-contaminated zirconia.^1,8,20
^


Nevertheless, most existing reports have evaluated either bond strength after decontamination or the underlying surface chemistry in isolation, rather than examining both endpoints, together with the effect of thermocyclic aging, within the same experimental model.^1,20
^ Consequently, it remains unclear whether the mechanical benefit of MDP-based cleaning persists under simulated long-term function, and whether it can be directly linked to a measurable chemical signature at the cement–zirconia interface.

This study aimed to evaluate the effect of an MDP-based cleaner (Katana Cleaner, Kuraray Noritake Dental, Tokyo, Japan) on the shear bond strength and chemical interaction of a self-adhesive resin cement (Panavia SA Cement Universal, Kuraray Noritake Dental, Tokyo, Japan) bonded to clean and blood-contaminated zirconia, both before and after thermocycling. Surface elemental composition and cement–zirconia chemical interactions were further characterized using X-ray photoelectron spectroscopy. The null hypotheses tested were that the MDP-based cleaner would not influence: (1) shear bond strength of the self-adhesive resin cement to zirconia; (2) the elemental composition of the treated zirconia surface; and (3) the chemical interaction between the resin cement and zirconia.

## Methods and Materials

The materials used in this study included an MDP-based cleaner (Katana Cleaner, Kuraray Noritake Dental, Tokyo, Japan) and a self-adhesive resin cement (Panavia SA Cement Universal, Kuraray Noritake Dental, Tokyo, Japan). An overview is presented in Table 1.

**Table 1 Table1:** Materials used in this study

Material	Batch number	Composition	Application
Katana Cleaner (Kuraray Noritake Dental, Tokyo, Japan)	1G0038	Water, methacryloyloxydecyl dihydrogen phosphate, hydrophobic aromatic dimethacrylate, triethanolamine, polyethyleneglycol, stabilizer, dyes	1. Apply and rub for 20 s. 2. Rinse the surface with water and dry.
Panavia SA Cement Universal (Kuraray Noritake Dental, Tokyo, Japan)	8R0268	Bisphenol A diglycidylmethacrylate, triethylene glycol dimethacrylate, 2-hydroxyethyl methacrylate, sodium fluoride, silanated barium glass filler, silanated colloidal silica, aluminum oxide filler, 10-methacryloyloxydecyl dihydrogen phosphate, hydrophobic aromatic dimethacrylate, silane coupling agent, dl-camphorquinone, peroxide, accelerators, catalysts, pigments	1. Apply through an automix tip. 2. Light cure for 5 s. 3. Remove the excess cement. 4. Light cure for 10 s.


### Zirconia Disc Preparation

One hundred and fifty-six cylindrical 5 mol% yttria-partially stabilized tetragonal zirconia polycrystalline (5Y-PSZ) specimens, measuring 12 mm in diameter and 5 mm in height, were pressed from the 5 mol% yttria-stabilized zirconia powder (Kuraray Noritake Dental, Tokyo, Japan) and sintered according to the manufacturer’s recommended firing protocol. All specimens then underwent standardized airborne-particle abrasion with 50-μm aluminum oxide particles, applied perpendicular to the bonding surface at 0.2 MPa for 20 s from a standardized distance of 10 mm. Air-abraded specimens were ultrasonically cleaned in 99% ethanol for 3 min and dried with oil- and moisture-free air.

### Shear Bond Strength (SBS)

One hundred and twenty zirconia discs were randomly allocated to four experimental groups for shear bond-strength testing. The control group comprised clean zirconia specimens receiving no pretreatment. In the MDP-based cleaner group, specimens were treated with the MDP-based cleaner for 20 s followed by a 10-s water rinse. In the blood-contaminated group, specimens were contaminated with 20 μL of sheep blood applied to the zirconia surface, left undisturbed for 60 s, and rinsed with water for 10 s. The blood-contaminated/MDP-based cleaner group underwent blood contamination, followed by MDP-based cleaner treatment as described for the second group. All specimens were air-dried for 10 s after their respective treatments. The experimental design is illustrated in Figure 1.

**Fig 1 Fig1:**
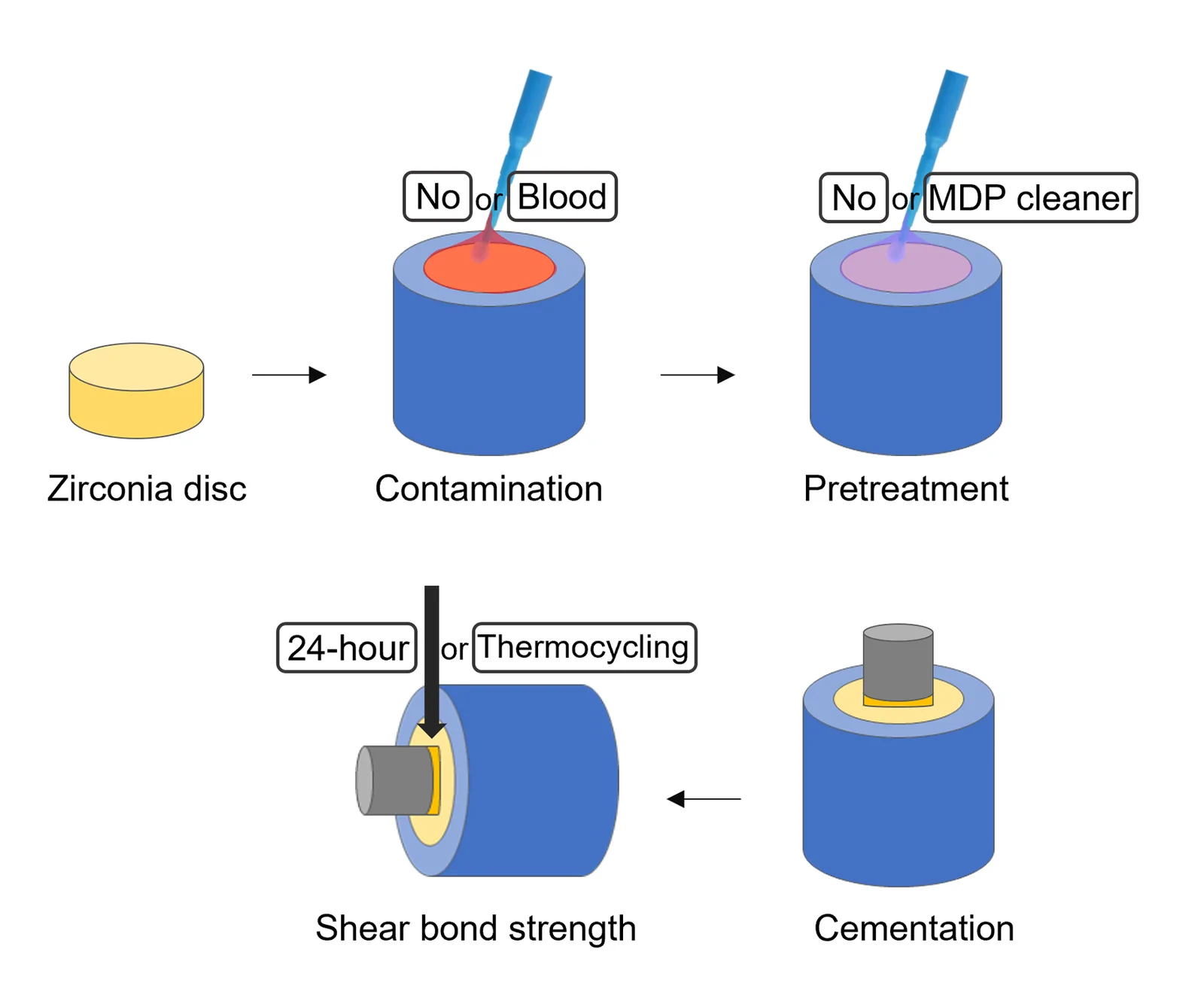
Schematic illustration of the experimental design for shear bond strength testing.

A custom plastic adhesive sheet with a centrally located, 2-mm-diameter circular aperture was positioned on each zirconia specimen to standardize the bonding area, after which the contamination and pretreatment protocols were applied as above. Following air-drying, self-adhesive resin cement was applied to the surface of a cylindrical metal rod (2-mm diameter × 5-mm height), which was then apposed precisely to the prepared zirconia surface. Initial photopolymerization was performed for 5 s (1,000 mW/cm,^3^ Valo Grand-Cordless, Ultradent, South Jordan, UT, USA) from two opposing directions; excess cement was removed with a hand scaler, and a further 10-s cure was applied from two opposing directions. Bonded assemblies were stored in deionized water at 37°C for 24 h; the adhesive plastic sheet was removed only after this storage period to avoid disturbing the bond interface before complete polymerization.

SBS was tested immediately after 24-h storage or after 5,000 thermocycles between 5°C and 55°C water baths, with a 30-s dwell time in each bath and a 5-s transfer time.^5^ Shear bond strength was measured using a universal testing machine (Instron 8872, Instron, High Wycombe, UK) at a crosshead speed of 1 mm/min.

The Kolmogorov–Smirnov and Levene’s tests confirmed that the SBS data were normally distributed and had homogeneous variance, respectively. Data were analyzed with three-way ANOVA (variables: contamination, MDP-based cleaner, and aging) followed by Tukey’s post hoc test. Bond durability, defined as the difference in SBS between 24-h storage and thermocycling with each group, was analyzed with independent-samples t-tests. All analyses were performed at α = 0.05 using SPSS (version 29.0.1, IBM, Chicago, IL, USA).

### Fractography

Fracture surfaces were examined under a stereomicroscope (Olympus SZ61, Tokyo, Japan) at 20× magnification. Failure mode was categorized as cohesive failure in resin cement, adhesive failure at the zirconia–resin cement interface, or mixed failure (a combination of adhesive and cohesive failure). The distribution of failure modes was analyzed using Pearson’s chi-square test at α = 0.05.

### Surface Morphology of Pretreated Zirconia

Twelve zirconia specimens were allocated to four groups (n = 3): (1) clean zirconia, (2) clean zirconia treated with MDP-based cleaner, (3) blood-contaminated zirconia, and (4) blood-contaminated zirconia treated with MDP-based cleaner. Specimens were desiccated for 24 h, sputter-coated with gold, and examined by scanning electron microscopy (SEM; Quanta 250, FEI, Hillsboro, OR, USA) at 3,000× magnification. The experimental design is illustrated in Figure 2.

**Fig 2 Fig2:**
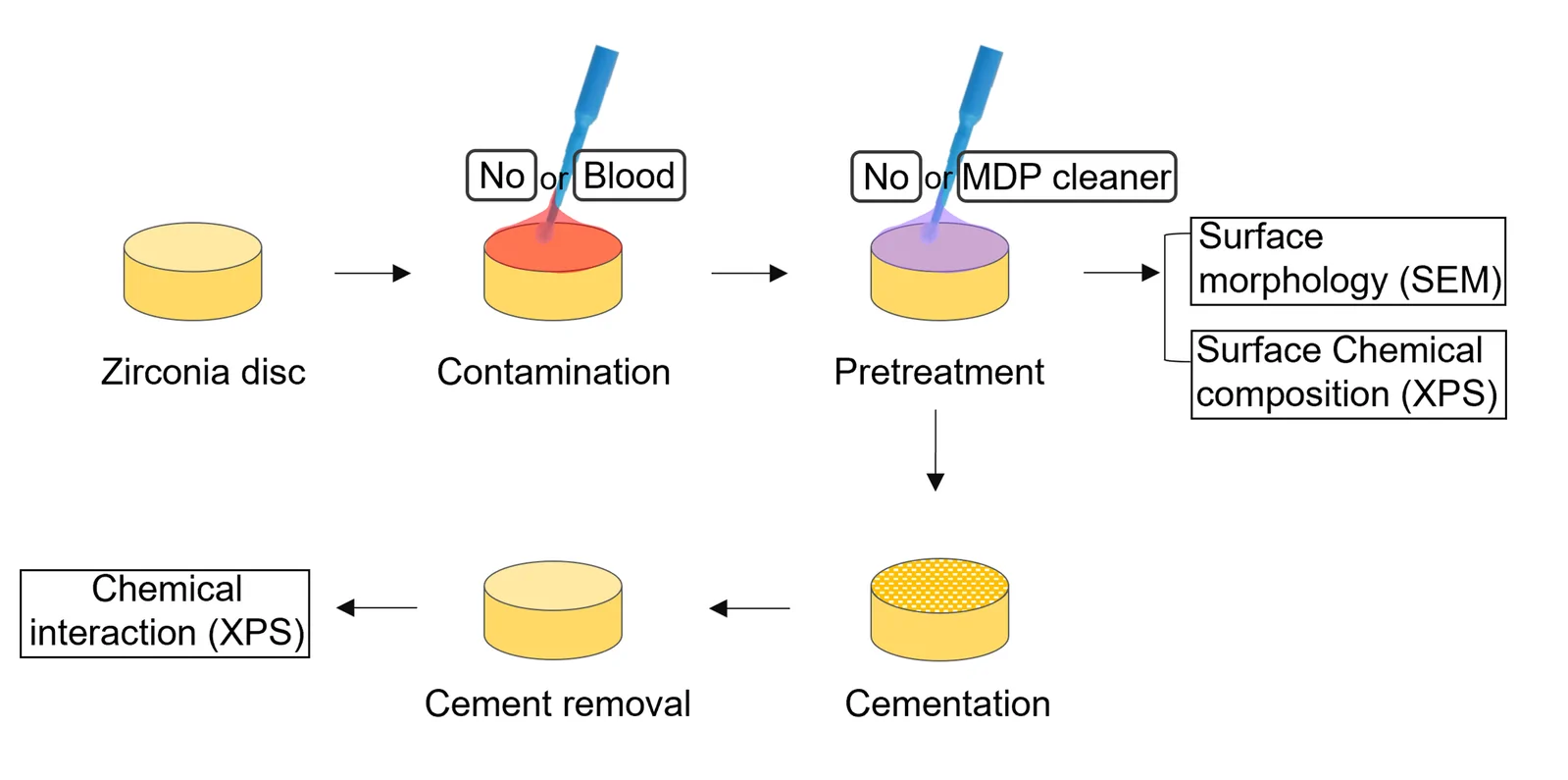
Schematic illustration of the experimental design for surface chemical composition and cement–zirconia chemical interaction analyses.

### Surface Chemical Composition of Pretreated Zirconia

Twelve zirconia specimens were allocated to the same four groups (n = 3) described earlier. Following their respective treatments, specimens were dried with oil-free compressed air, and elemental composition was characterized by X-ray photoelectron spectroscopy (XPS; Kratos Axis Ultra DLD, Kratos Analytical, Manchester, UK). Analyses used monochromatic Al Kα radiation (hν = 1486.8 eV) generated at 15 kV and 10 mA (180 W total power). Photoelectrons were detected within a 700 × 300 μm analytical area under ultra-high vacuum (base pressure 3 × 10^−9^ Torr) using a 180° hemispherical energy analyzer with five channeltron detectors. Survey spectra were acquired at a pass energy of 1000 meV with a 100-ms dwell time per increment. The experimental design is illustrated in Figure 2.

### Chemical Interaction of Self-adhesive Resin Cement

Twelve zirconia specimens were allocated to the same four groups (n = 3) described above. Self-adhesive resin cement was applied to the treated zirconia surfaces and light-cured for 20 s using an LED light-curing unit (1,000 mW/cm,^3^ Valo Grand-Cordless, Ultradent, South Jordan, UT, USA). After curing, the cement was carefully removed from the zirconia surfaces using a clean blade without damaging the underlying substrate, and the exposed surfaces were analyzed by XPS as described earlier. The experimental design is illustrated in Figure 2.

## Results

### Shear Bond Strength

Shear bond strength values are presented in Table 2. Three-way ANOVA showed that SBS was significantly influenced by contamination (*p* < 0.001), MDP-based cleaner (*p* < 0.001), and thermocycling (*p* = 0.047), with a significant interaction between contamination and MDP-based cleaner (*p* < 0.001); no other interactions were significant (*p* > 0.05).

**Table 2 Table2:** Means and standard deviations of shear bond strength (MPa) and failure mode distribution (number of specimens)

Substrate	Pretreatment	24 h Mean ± SD (A/M/R/Z)	Thermocycling Mean ± SD (A/M/R/Z)
Clean zirconia	None	30.4 ± 5.5^Aa^ (2/8/5/0)	29.0 ± 5.3^Aa^ (2/9/4/0)
	MDP-based cleaner	31.3 ± 3.6^Aa^ (0/10/5/0)	31.3 ± 7.1^Aa^ (0/11/4/0)
Blood-contaminated zirconia	None	21.9 ± 3.3^Ba^ (8/5/2/0)	16.9 ± 4.2^Bb^ (8/6/1/0)
	MDP-based cleaner	31.7 ± 4.2^Aa^ (3/8/4/0)	29.4 ± 4.3^Aa^ (3/9/3/0)
Different uppercase letters indicate statistically significant differences within each column (Tukey’s post hoc test, *p* < 0.001); different lowercase letters indicate statistically significant differences within each row (independent t-test, *p* < 0.05). A: adhesive failure; M: mixed failure; R: cohesive failure in resin cement; Z: cohesive failure in zirconia.			

For clean zirconia, neither MDP-based cleaner pretreatment nor thermocycling significantly influenced SBS (*p* > 0.05), and bond strength remained stable after thermocycling in both groups (*p* > 0.05). For blood-contaminated zirconia, the untreated group showed significantly lower bond strength than all other groups at 24 h (*p* < 0.001), which decreased further after thermocycling (*p* = 0.004). Notably, MDP-based cleaner application to contaminated zirconia substantially improved initial bond strength (*p* < 0.001) and this improvement remained stable after thermocycling (*p* = 0.12).

### Fractography

The distribution of failure modes across all experimental groups is presented in Table 2. Representative fractographs are shown in Figure 3. Mixed failure was the predominant mode in all groups. The contaminated zirconia groups showed a higher proportion of adhesive failures; however, this difference did not reach statistical significance among the experimental groups (*p* = 0.16).

**Fig 3 Fig3:**
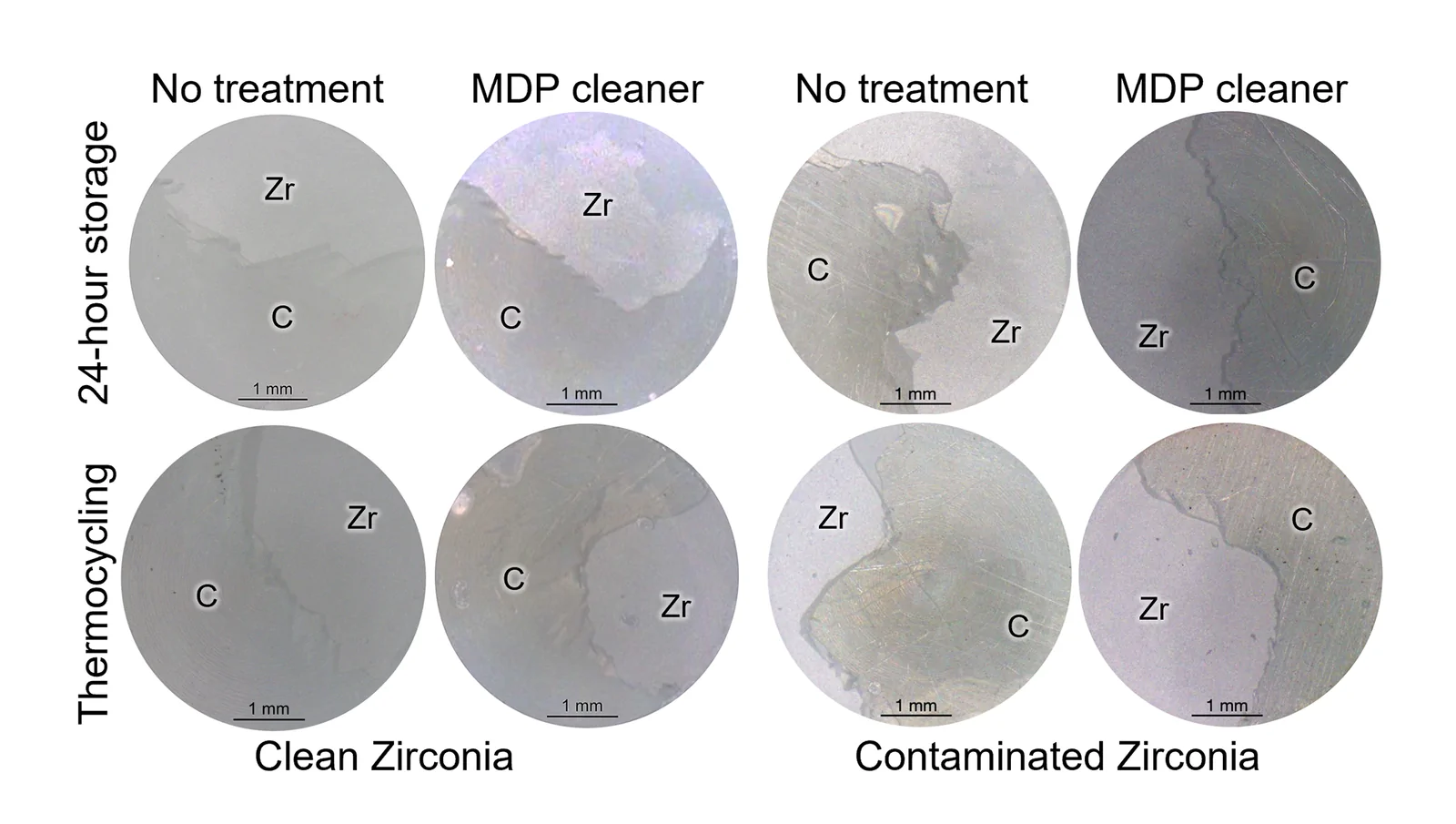
Representative fractography. C: resin cement; Zr: zirconia.

### Surface Morphology

Representative SEM images of zirconia surfaces under the four treatment conditions are shown in Figure 4. Micrographs revealed similar surface topographies across all groups, regardless of contamination status or cleaner application. Clean, untreated zirconia (Fig 4a) exhibited a characteristic polycrystalline microstructure with visible grain boundaries and crystalline facets. Application of the MDP-based cleaner to clean zirconia (Fig 4b) did not alter this morphology. Blood contamination (Fig 4c) likewise produced no discernible microscopic change compared with clean surfaces, and this remained the case when contaminated surfaces were subsequently treated with the MDP-based cleaner (Fig 4d).

**Fig 4a to d Fig4atod:**
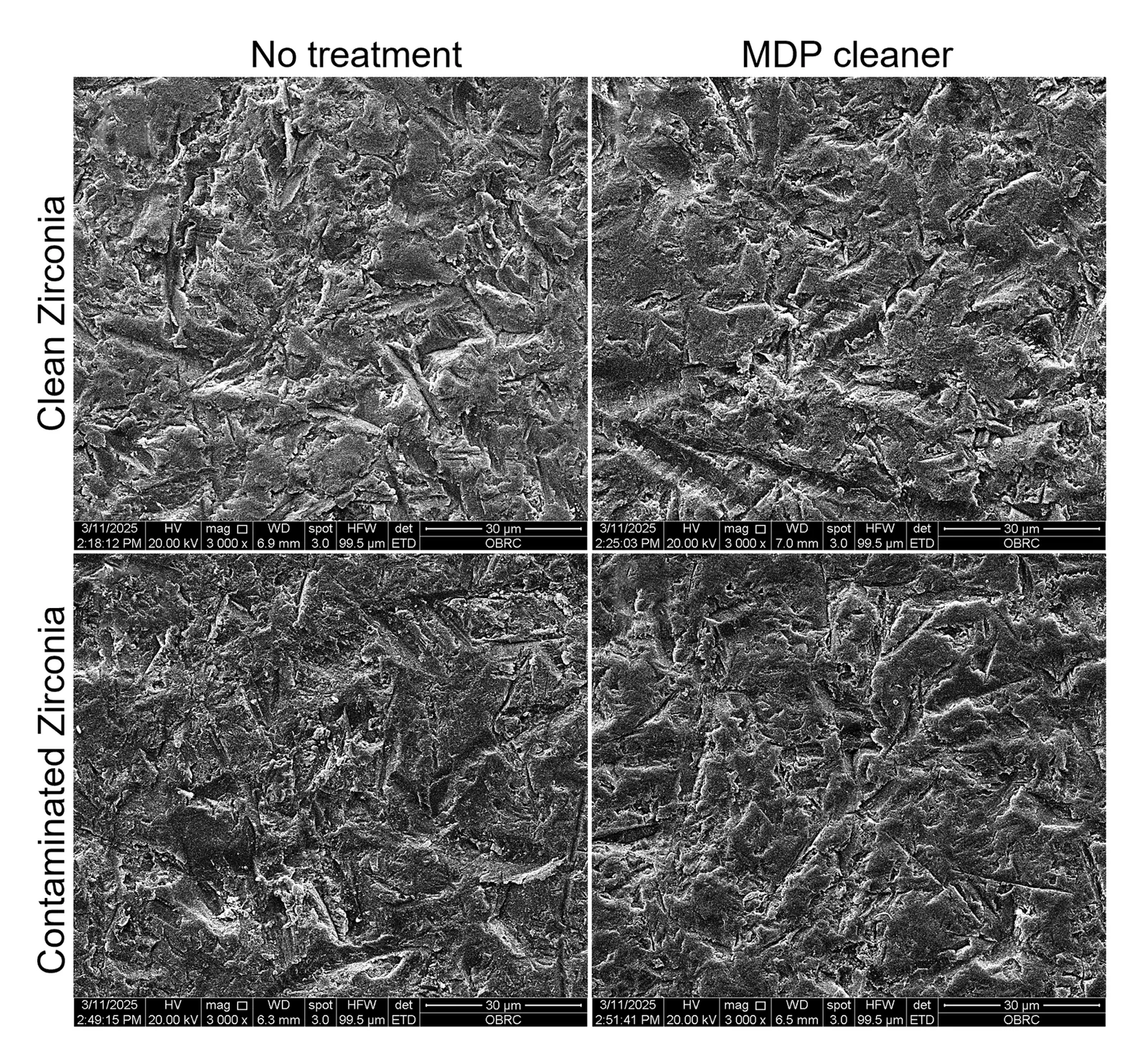
Representative SEM images (3,000×) of zirconia surfaces: (a) clean zirconia; (b) clean zirconia treated with MDP-based cleaner; (c) blood-contaminated zirconia; (d) blood-contaminated zirconia treated with MDP-based cleaner.

### Surface Chemical Composition

XPS spectra revealed distinct surface chemical compositions across the four zirconia surface treatments (Fig 5). Clean zirconia exhibited characteristic peaks for O1s (530.1 eV), C1s (284.8 eV), Zr3p (333.1 eV), and Zr3d (182.3 eV) (Fig 5a). After MDP-based cleaner treatment, a prominent P2p peak emerged at 133.5 eV (Fig 5b), indicating the presence of phosphate-containing species derived from MDP on the zirconia surface.

**Fig 5a to d Fig5atod:**
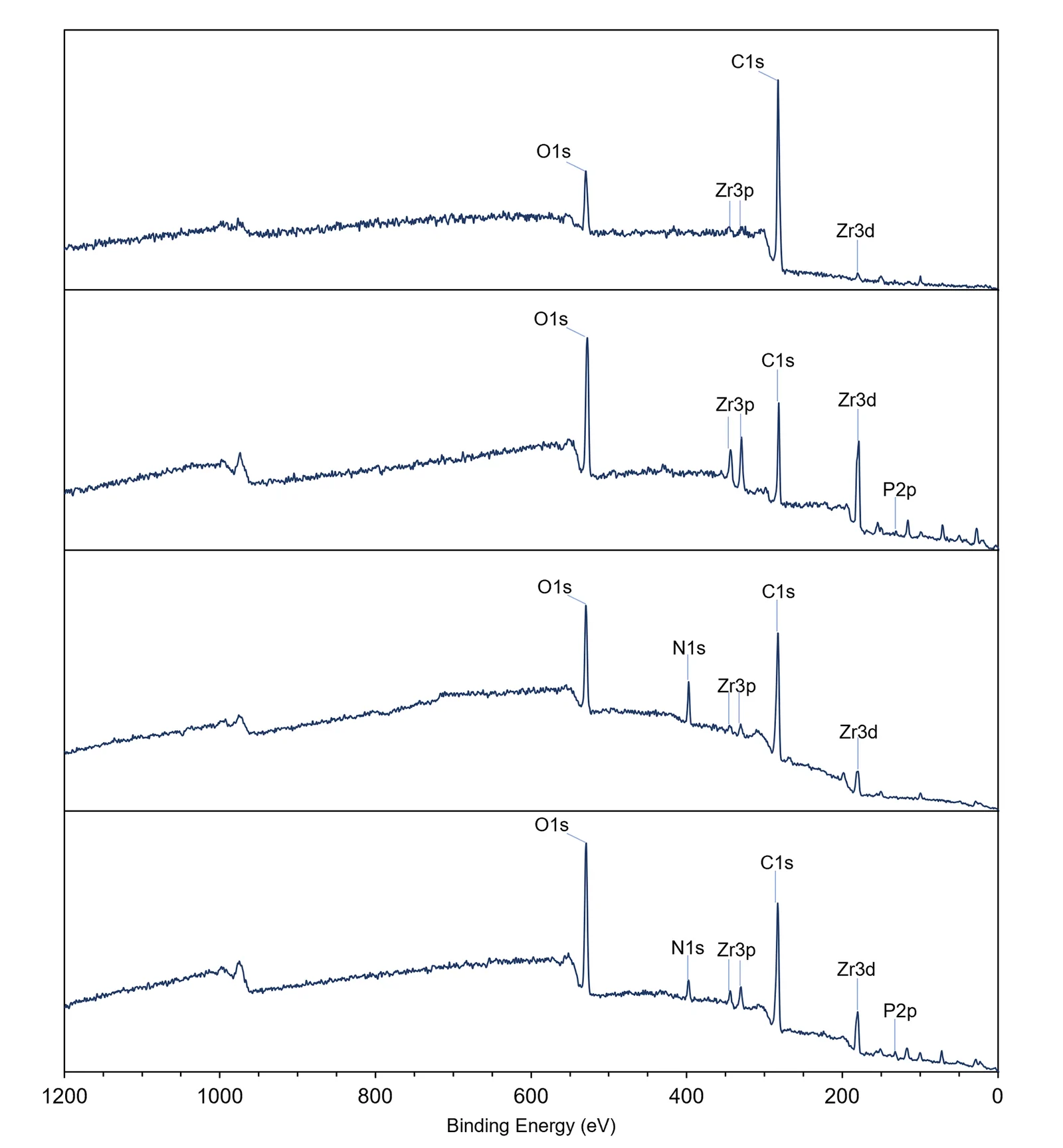
XPS survey spectra of zirconia surfaces: (a) clean zirconia; (b) clean zirconia treated with MDP-based cleaner; (c) blood-contaminated zirconia; (d) blood-contaminated zirconia treated with MDP-based cleaner.

Blood-contaminated zirconia displayed a characteristic N1s peak at 399.8 eV – attributable to peptide bonds and amino groups in blood proteins – together with increased C1s intensity and attenuated Zr3d and Zr3p signals (Fig 5c). Following MDP-based cleaner application to contaminated surfaces, both N1s and P2p peaks were detected, with N1s intensity reduced relative to the untreated contaminated sample, and Zr3d signal intensity increased relative to the contaminated surface (Fig 5d). This spectral profile suggests a dual mechanism: partial removal of nitrogenous contaminants coupled with integration of phosphate groups from the MDP-based cleaner onto the partially re-exposed zirconia surface.

### Chemical Interaction

XPS analysis of the zirconia surfaces after cementation and subsequent cement removal revealed distinct chemical signatures reflecting the underlying substrate treatment (Fig 6). For clean zirconia, cementation produced characteristic O1s (530.1 eV) and C1s (284.8 eV) peaks together with marked attenuation of the Zr3p and Zr3d signals relative to the pristine surface (Fig 5a), and a concurrent increase in O1s intensity (Fig 6a). This spectral pattern supports a chemical interaction between MDP-derived phosphate groups and zirconia. Clean zirconia pretreated with the MDP-based cleaner showed an essentially identical response, with attenuated Zr3p/Zr3d peaks and increased O1s intensity relative to the corresponding pre-cementation spectrum (Figs 5b vs 6b), suggesting that the cleaner did not interfere with subsequent interaction of the cement with zirconia.

**Fig 6a to d Fig6atod:**
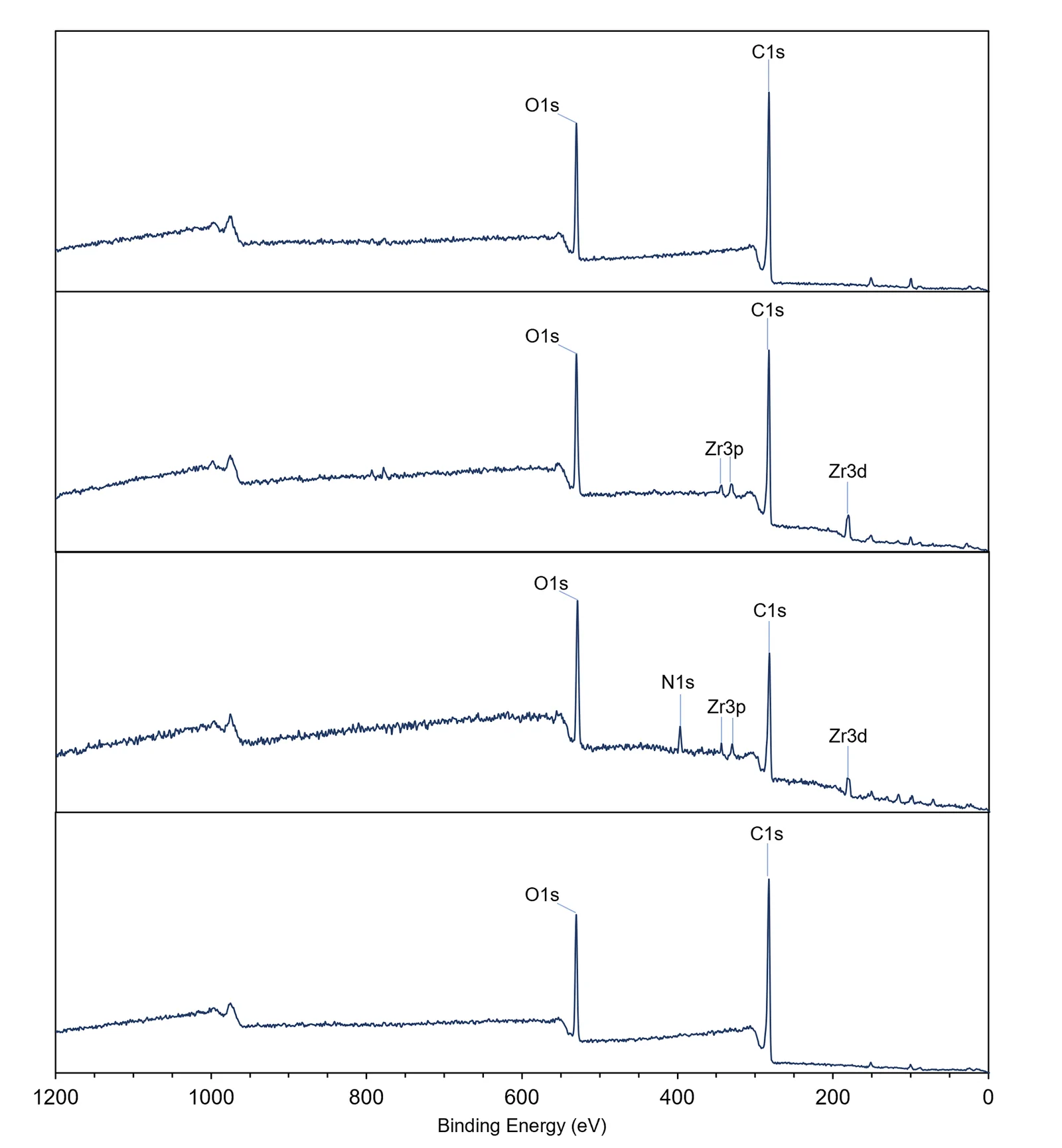
XPS spectra of zirconia surfaces after cementation with self-adhesive resin cement and cement removal: (a) clean zirconia; (b) clean zirconia treated with MDP-based cleaner; (c) blood-contaminated zirconia; (d) blood-contaminated zirconia treated with MDP-based cleaner.

In contrast, blood-contaminated zirconia retained a prominent N1s peak after cementation, and Zr3p and Zr3d signal intensities remained essentially unchanged from the pre-cementation spectrum (Figs 5c vs 6c), despite an O1s intensity comparable to the clean groups. This pattern indicates that adsorbed blood proteins persisted at the interface and sterically blocked direct coordination between the cement’s phosphate groups and the zirconium oxide surface. When contaminated zirconia was treated with the MDP-based cleaner before cementation, the resulting spectrum closely resembled that of clean zirconia: N1s intensity was markedly reduced and Zr3p/Zr3d attenuation was restored (Fig 6d), indicating that direct cement–zirconia chemical bonding had been re-established. These chemical findings parallel the shear bond strength results and provide a mechanistic explanation for the recovery of bond strength observed in MDP-based cleaner–treated contaminated specimens.

## Discussion

MDP-based cleaner treatment significantly improved the shear bond strength of the resin cement to blood-contaminated zirconia compared with untreated contaminated surfaces, and this improvement was maintained after thermocycling. SEM showed no morphological alterations across treatment groups, indicating that the improved bond performance resulted from chemical rather than topographical modification, while XPS revealed distinctive chemical changes: the emergence of P2p signals after MDP-based cleaner application indicated successful phosphate attachment to zirconia, and reduced N1s intensity in contaminated specimens indicated partial protein removal. Analysis of the surfaces recovered after cementation further indicated that treatment with MDP-based cleaner restored a surface chemistry consistent with phosphate-zirconia interaction even on previously contaminated surfaces. On the basis of these findings, all three null hypotheses were rejected: the MDP-based cleaner significantly influenced (1) bond performance of the self-adhesive resin cement, particularly to contaminated zirconia; (2) surface elemental composition, evidenced by the presence of phosphate groups and reduction of nitrogenous contaminants; and (3) the chemical interaction between the resin cement and zirconia, evidenced by the persistence of chemical signatures after cementation.

SEM evaluation demonstrated that neither blood contamination nor MDP-based cleaner application produced discernible alterations to zirconia surface microstructure, supporting the conclusion that the bond-strength benefit of MDP-based cleaner treatment derives primarily from chemical rather than topographical modification. This observation is consistent with previous reports that MDP-containing conditioning agents do not measurably alter zirconia surface topography.^1,15
^


XPS analysis provided supporting evidence for the specific chemical mechanism underlying the action of the MDP-based cleaner. The emergence of P2p signals after MDP-based cleaner application indicates uptake of phosphate groups from MDP onto the zirconia surface. This observation is compatible with the established model in which 10-MDP interacts with zirconia predominantly through Zr–O–P coordination complexes, with phosphate groups coordinating directly to zirconium atoms at the substrate surface.^12^ The survey spectra acquired in the present study, however, do not resolve the chemical states that would be required to demonstrate Zr–O–P bond formation directly. The concurrent increase in O1s intensity following MDP-based cleaner application further corroborates this interaction mechanism.

Particularly noteworthy was the finding that the MDP-based cleaner established a phosphorus signal on the zirconia surface even in the presence of blood contamination. While blood introduced nitrogen-containing species that partly masked the zirconia surface,^11,12
^ subsequent MDP-based cleaner application reduced N1s intensity while simultaneously establishing P2p signals—indicating that the cleaner partially removed or displaced blood proteins while phosphate was concurrently taken up on the underlying substrate. This dual functionality helps explain the superior effectiveness of MDP-containing cleaners, relative to conventional decontamination methods such as water rinsing or phosphoric-acid etching, in recovering bond strength to contaminated zirconia.^1,3,6
^


Analysis of Zr3d and O1s peak intensities across treatment groups provided further insight into the mechanism of MDP-based cleaner action. The increased intensity of these peaks after cleaner application to contaminated surfaces indicates improved access to the underlying zirconia substrate, suggesting that the cleaner not only support the formation of chemical bonds but also enhances surface wettability by reducing protein coverage.^9,15
^ Improved wettability would, in turn, facilitate more effective interaction with subsequently applied resin cement, offering a plausible explanation for the enhanced bond strength observed in mechanical testing.

Nonetheless, the persistence of an attenuated N1s peak in blood-contaminated samples after MDP-based cleaner application indicates incomplete removal of blood proteins. This partial-cleaning effect suggests that, while MDP-cleaners significantly improve bond strength to contaminated zirconia, optimal results in severely contaminated clinical scenarios might require additional or complementary cleaning procedures.^6,18
^


These findings suggest that MDP-based conditioning agents can restore adhesive bond strength to contaminated zirconia and offer clinicians an evidence-based approach for managing inadvertent contamination during prosthetic try-in. The bifunctional mechanism – a partial reduction of surface contamination combined with phosphate uptake on the zirconia surface – represents an efficient clinical protocol, particularly advantageous where optimal field isolation is difficult to achieve, and the demonstrated thermocycling stability of MDP-conditioned specimens suggests a potential benefit for the long-term durability of zirconia-based prosthetic restorations.^2,16,17
^


Certain limitations should be acknowledged. Bond strength was assessed with a macro-shear test, in which the interfacial stress field is non-uniform and concentrated at the loading edge.^3,4
^ The chemical characterization rests on XPS spectra; high-resolution narrow scans of the P2p, O1s and Zr3d regions, which would be required to resolve the chemical states of phosphorus and oxygen and thereby identify a Zr–O–P coordination environment, were not acquired. The present findings should therefore be read as evidence of phosphate uptake and of an interaction between MDP-derived phosphate and zirconia, rather than as direct proof of a defined coordination bond, and confirmation by high-resolution XPS of the P2p, O1s, and Zr3d regions. Although thermocycling provides valuable insight into bond durability, it represents only one dimension of the multifactorial intraoral aging process, and the temperature and cycle parameters used here approximate, rather than fully replicate, clinical conditions.^5^
*In vitro* conditions also cannot fully capture the complexity of the oral environment, including variability in isolation protocols and operator technique. Notwithstanding these constraints, the present findings carry meaningful clinical implications for the management of contaminated zirconia restorations.

## Conclusion

Within the limitations of this *in vitro* study, the MDP-based cleaner had a negligible effect on the bond performance of self-adhesive resin cement to clean zirconia but significantly enhanced adhesion to blood-contaminated surfaces and maintained bond stability after thermocycling. This efficacy can be attributed to a bifunctional mechanism combining reduction of surface contamination with chemical bond formation. These properties offer clinicians an efficient approach for managing inadvertent contamination during prosthetic procedures, contributing to more predictable clinical outcomes for zirconia restorations under challenging isolation conditions.

### Acknowledgments

#### Funding statement (open access)

This study was funded by the Faculty Research Grant, Faculty of Dentistry, Chulalongkorn University. J. Tagami is supported by the High-Potential Professors, the Second Century Fund (C2F), Chulalongkorn University.

#### Clinical relevance

Inadvertent blood contamination during clinical try-in can compromise the resin bond to zirconia restorations. Decontaminating the fitting surface with an MDP-based cleaner before cementation restores shear bond strength to the level recorded on uncontaminated zirconia and re-establishes the surface phosphate uptake associated with chemical interaction, offering clinicians a simple chairside method to safeguard adhesive performance.

#### Ethical statement

The sheep blood used for the contamination protocol was obtained as a slaughterhouse by-product and did not require institutional animal ethics committee approval.

#### Conflict of interest

The authors declare no conflict of interest.

#### Data availability

The data that support the findings of this study are available from the corresponding author on reasonable request.

#### AI use declaration

During the preparation of this manuscript, the authors used an AI-based language model (Claude, Anthropic) to assist with language editing. All AI-assisted editing was reviewed and approved by the authors.

## References
